# Interaction between demand-control and social support in the occurrence of common mental disorders

**DOI:** 10.1590/S1518-8787.2017051006446

**Published:** 2017-05-08

**Authors:** Amália Ivine Santana Mattos, Tânia Maria de Araújo, Maura Maria Guimarães de Almeida

**Affiliations:** I Programa de Pós-Graduação em Saúde Coletiva. Universidade Estadual de Feira de Santana. Feira de Santana, BA, Brasil; II Departamento de Saúde. Universidade Estadual de Feira de Santana. Feira de Santana, BA, Brasil

**Keywords:** Occupational Health, Mental Health, Working Conditions, Job Satisfaction, Burnout, Professional, epidemiology, Mental Disorders, epidemiology, Social Support, Job Strain Model

## Abstract

**OBJECTIVE:**

To analyze the interaction between the psychosocial aspects of work and the occurrence of common mental disorders among health workers.

**METHODS:**

This is a cross-sectional study conducted with a representative sample of workers of the primary health care of five municipalities of the State of Bahia, Brazil, in 2012. The variable of outcome were the common mental disorders evaluated by the SRQ-20, and the variables of exposure were high demand (high psychological demand and low control over the work) and low social support in the workplace. Interaction was checked by the deviation of the additivity of the effects for the factors studied from the calculation of excess risk from interaction, proportion of cases attributed to interaction, and the synergy index.

**RESULTS:**

The global prevalence of common mental disorders was 21%. The group of combined exposure has shown higher magnitude (high demand and low social support), reaching 28% when compared to the 17% in the situation of no exposure (low demand and high social support).

**CONCLUSIONS:**

The results strengthen the hypothesis of interaction between the factors investigated, directing to the synergy of the effects.

## RESUMO

**OBJETIVO:**

Analisar a interação entre aspectos psicossociais do trabalho e a ocorrência de transtornos mentais comuns entre trabalhadores da saúde.

**MÉTODOS:**

Estudo transversal conduzido em amostra representativa de trabalhadores da atenção básica de cinco municípios da Bahia em 2012. As variáveis desfecho foram os transtornos mentais comuns avaliados pelo SRQ-20, as de exposição foram a alta exigência (alta demanda psicológica e baixo controle sobre o próprio trabalho) e o baixo apoio social no trabalho. A interação foi verificada pelo afastamento da aditividade dos efeitos para fatores estudados a partir do cálculo do excesso de risco devido à interação, proporção de casos atribuída à interação e índice de sinergia.

**RESULTADOS:**

A prevalência global de transtornos mentais comuns foi de 21%. Apresentou maior magnitude no grupo de exposição combinada (alta exigência e baixo apoio social), chegando a 28% quando comparada a 17% na situação de nenhuma exposição (baixa exigência e alto apoio social).

**CONCLUSÕES:**

Os resultados fortalecem a hipótese de interação entre os fatores investigados, com direção para a sinergia dos efeitos.

## INTRODUCTION

The scientific literature points to the influence that work has on the lives of individuals^18^ ^,^ ^19^. Work can also contribute to change the physical and mental health of individuals, since it involves complex articulations of factors involving the body and the subjectivity of workers^12^. Work experiences that allow the individual to use fully their skills, express their creativity, and control significantly their work are reported as promoters of achievement, pleasure, health, and satisfaction^15^. On the other hand, the work developed under conditions that offer low control, with high psychological demands, marked by conflicting social relations, or characterized by social isolation (isostrain) may constitute an important factor for mental illness^2^.

Mental disorders related to work reach a significant portion of the Brazilian working population. They are responsible for the third place in the ranking related to the granting of sickness benefits^25^. Common mental disorders (CMD), of high occurrence among health workers, have been observed as a factor associated with presenteeism, absenteeism, and disabilities^22^. Their occurrence among health workers is a problem of great relevance, because this implies impairment of the care provided to the user of the health system^1^.

Among the stressful events capable of causing CMD, we can mention the psychosocial aspects of work, which can be measured by the demand-control model, proposed by Karasek^17^. The model allows for the prediction of four work experiences: low demand (high control/low demand), active work (high control/high demand), passive work (low control/low demand), and high demand (low control/high demand). Work experiences that combine high psychological demands and low control over the work offer increased job strain and, consequently, deleterious effects on the health of workers^17^.

Although suitable, the demand-control model was considered limited to grasp the complexity of the work process and its relationship with health outcomes. In this way, a third dimension was incorporated into the demand-control model: social support in the workplace. The inclusion of this third psychosocial aspect has been proposed by Johnson^16^, when considering that social support, a basic human need, would act as an important moderator of the impacts of high demands, what he has called as collective control. Social support refers to the proposal of a defensive ideology, since it consists in adopting collective strategies for the management of the hardships experienced in the work environment^12^.

Several authors have demonstrated that these three psychosocial dimensions of work – psychological demand, decision latitude, and social support – act as factors that interfere with the occurrence of outcomes related to health^6^. Thus, it is pertinent to consider the existence of an interaction between these dimensions, since this interaction is characterized by the deviation of the additivity of individual effects^21^. In the presence of two or more factors, the combined action differs from the simple act of each exposure separately, either for synergy or antagonism.

The study of the variability of diseases in human populations is one of the main objectives of epidemiology. However, the explanations for such variability are not easy to show, because explanatory models able to characterize how exposure contributes to the development of diseases are still hard to find in this field of knowledge. The evaluation of the interaction of factors for the occurrence of diseases is a privileged analysis alternative since, in a complex network of interaction of causes, interference in any of the factors that make it can deconstruct the entire framework responsible for generating the disease^21^.

There are few studies that have investigated the interaction between psychosocial aspects, considering the decision latitude, psychological demand, and social support in the workplace, and the occurrence of outcomes related to mental health^8^. We need to understand the complex mechanisms of interaction between these three psychosocial dimensions that trigger common mental disorders, since this knowledge can represent relevant gains toward the prevention of this issue among workers. The objective of this study has been to analyze the interaction between the psychosocial aspects of work (psychological demand, decision latitude, and social support) and the occurrence of CMD among health workers.

## METHODS

This is a cross-sectional study conducted in five municipalities of the State of Bahia, Brazil, with health workers focusing on working conditions, employment, and health. We included service workers of the primary health care who were in full exercise of their activities. The representative sample was selected randomly, stratified by geographic area, level of complexity of the care, and occupational group. The data on the workforce of the units were supplied by the municipal departments of health of the municipalities studied. We selected the workers to participate in the study using lists of random numbers.

Data collection involved application of a research instrument constructed from the literature review. Interviewers were previously trained to standardize behaviors and a pilot study was carried out in a municipality of Bahia with 30 health workers. The data were collected in 2012 from visits to health units where the selected professionals worked. We used the strategy of replacing a professional of the same gender and professional category after three unsuccessful attempts to contact, as a procedure to reduce possible losses.

The variable of outcome was the CMD measured by the Self-Reporting Questionnaire (SRQ-20), an instrument composed of 20 dichotomous questions (yes, no) that evaluate the depressive, somatic, and anxiety symptoms that occurred in the last 30 days. In the evaluation carried out by Santos et al.^23^, the SRQ-20 obtained an acceptable performance to assess the mental health of workers in Bahia. The cutoff point for suspected CMD was seven or more positive responses. This cutoff point was considered as adequate in discriminating suspected cases in a study conducted with urban population^24^.

The variables of exposure were measured by the version translated to Portuguese of the Job Content Questionnaire (JCQ). The instrument is composed of 49 questions, distributed into five dimensions: physical demands, psychological demands, decision latitude, social support in the workplace, and job insecurity. In this study, the dimensions used were: psychological demands, decision latitude, and social support in the workplace. The questions of the JCQ are placed in a four-point ordinal Likert-type scale (“strongly disagree” to “strongly agree”). A study on the performance of the JCQ has shown that it is a good instrument to measure the psychosocial aspects of the work in Brazil^5^.

The score obtained for each dimension was categorized into high and low using the average as cutoff point^4^. After this procedure, we set the quadrants of the work situations provided for by the model: low demand, active work, passive work, and high demand. Low social support in the workplace and high demand were regarded as the most harmful categories of exposure to the health of workers.

The covariates considered in the analysis were: socio-demographic information (gender, age, number of children, marital status, education level, income), work characteristics (type of employment, weekly work, time working in the unit, labor rights, double employment, compatibility of the activities developed, job satisfaction, housework, excessive housework), and life habits (physical activities, leisure activities). Excessive housework was obtained by calculating: SD = (washing + ironing + cleaning + cooking) x (number of residents - 1)^27^. The score obtained was categorized in tertiles.

The analysis of the interaction involved the construction of dummy variables to establish the exposures: no exposure to any of the factors = high social support, low demand (R_00_), independent exposures = high social support, high demand (R_01_), low social support, low demand (R_10_), and combined exposure = low social support, high demand (R_11_). The measures of interaction based on the additivity criterion were verified by calculating the excess risk due to interaction (excess risk due to interaction – RERI = PR_11_ - PR_01_ - PR_10_ + 1), which quantifies the deviation from the null value, and the attributable proportion due to interaction (attributable proportion due to interaction – AP = (PR_11_ - PR_01_ - PR_10_ + 1) / PR_11_), which shows the proportion of cases from both exposures and synergy index (S = (PR_11_ - 1) / (PR_01_ + PR_10_ - 2)) that reflects the direction of the interaction in relation to nullity (S = 1), synergy (S > 1), or antagonism (S < 1)^21^. We also calculated excess of prevalence (excess of prevalence – EP = P_exposure_ - P_no exposure_) and excess of prevalence ratio (EPR = PR - 1), which show whether the combined effect of the factors is greater than the sum of their individual effects, and relative difference [(PR - 1 / EPR_01_ + EPR_10_) - 1], which shows the deviation of the expected behavior for the isolated action of the factors^14^.

The research project has been approved by the Research Ethics Committee of the *Universidade Estadual de Feira de Santana* (Protocol 081/2009). All phases of this study have met Resolution 466/12 of the Brazilian National Health Council, which addresses the guidelines and standards that regulate research with humans. All interviewees signed the informed consent.

## RESULTS

The study population consisted of 2,532 young workers (average of 30.8 years), with female predominance (79.3%), married and in a stable union (57.7%), with high school (39.4%), brown (56%), and who practiced physical (70.4%) and leisure (81.9%) activities. Effective employment was referenced by 70.4% of the workers. Most claimed to have weekly workload of 40 hours (81.9%), only one employment (78.7%), time working in the unit of up to four years (51%), only partial labor rights (62.3%), and compatibility of the activities developed with the position (97.3%). Job satisfaction was reported by 73.9% of the workers. The workers predominantly had high psychological demand (54%), low decision latitude (59.4%), and high social support (54.2%). Work in high demand was experienced by 32.5% of the workers (data not shown in tables). Workers who experienced high demand presented greater percentages among those who had no partner (45.5%), did no physical (51.7%) or leisure (19.1%) activities, were dissatisfied with their work (35.7%), and had high excessive housework (32.9%). The prevalence of CMD was higher in this group (26.2%), suggesting that situations of greater vulnerability were concentrated in situations of high demand. Workers with low social support presented high percentages among males (20.4%), who were 40 years old or more (41%), with higher education (39.7%), with income greater than two minimum wages (25.1%), who did not practice physical (52.4%) or leisure (19.9%) activities, with no effective employment (30.8%), working up to four years in the unit (49.4%), dissatisfied with the work (34.6%), and with higher prevalence of CMD (24.4%) (Table 1).

**Table 1:** Characteristics of workers according to the demand-control model and social support in the workplace. State of Bahia, Brazil, 2012.

Variable	Exposure: demand-control model								Exposure: social support in the workplace			
	
	Low demand		Active work		Passive work		High demand		High support		Low support	
					
	n	%	n	%	n	%	n	%	n	%	n	%
Gender												
Female	369	81.3	375	74.6	506	80.4	612	80.2	1,034	80.8	859	79.6
Male	85	18.7	128	24.4	123	19.6	151	19.8	246	19.2	20	20.4
Age group (years)												
Up to 40	257	57.9	290	58.8	351	56.8	441	58.6	374	59.3	627	59.0
More than 40	187	42.1	203	41.2	267	43.2	312	41.4	511	40.7	435	41.0
Marital Status												
With partner	256	56.4	305	60.9	371	59.0	419	55.0	723	56.7	633	58.6
Without partner	198	43.6	196	39.1	258	41.0	343	45.0	553	43.3	447	41.4
Education level												
Up to high school	270	60.3	243	48.9	411	65.7	471	62.5	807	63.5	643	60.3
Higher education	178	39.7	254	51.1	215	34.3	283	37.5	464	36.5	424	39.7
Income												
Up to 2 minimum wages	274	76.5	228	61.3	388	84.9	471	82.5	829	82.5	586	74.9
More than 2 minimum wages	84	23.5	144	38.7	69	15.1	103	17.9	176	17.5	196	25.1
Physical activities												
Yes	235	52.5	286	57.9	329	52.8	366	48.3	695	55.0	509	47.6
No	213	47.5	208	42.1	294	47.2	391	51.7	569	45.0	560	52.4
Leisure activities												
Yes	391	86.1	434	87.0	507	81.3	615	80.9	1,076	84.9	864	80.1
No	63	13.9	65	13.0	117	18.8	145	19.1	191	15.1	214	19.9
Type of employment												
Effective	317	70.4	301	60.6	500	79.7	516	68.3	928	73.0	738	69.2
Not effective	133	29.6	196	39.4	127	20.3	239	31.7	343	27.0	329	30.8
Time working in the unit (years)												
Up to 4	227	51.6	213	44.3	325	54.2	350	47.4	584	48.4	511	49.4
More than 4	213	48.4	268	55.7	275	45.8	389	52.6	622	51.6	524	50.6
Satisfaction with the job												
Satisfied	384	84.6	399	79.3	451	71.8	489	64.3	1,033	80.8	706	65.4
Dissatisfied	70	15.4	104	20.7	177	28.2	272	35.7	245	19.2	374	34.6
Excessive housework												
Low	207	47.2	260	53.6	252	40.9	327	44.2	531	42.9	481	46.2
Average	98	22.3	96	19.8	164	26.6	169	22.9	290	23.5	247	23.8
High	134	30.5	129	26.6	200	32.5	243	32.9	416	33.6	312	30.0
CMD												
Yes	75	16.6	87	17.9	127	20.8	197	26.2	232	18.6	257	24.4
No	376	83.4	400	82.1	484	79.2	554	73.8	1,016	81.4	797	75.6

CMD: Common mental disorders

The global prevalence of CMD in the population studied was 21%. We observed the occurrence of CMD in 17% of those exposed to no factors and in 28% of those exposed to both factors. The combination of exposures presented a trend of increasing the crude prevalence ratios. The analysis of the effect of isolated factors showed that workers with high demand had higher prevalence of CMD than the reference group (PR = 1.41; 95%CI 1.02–1.94), as well as those who reported low social support in the workplace (PR = 1.05; 95%CI 0.67–1.62). Among those exposed to both high demand and low social support, we observed greater magnitude of association (PR = 1.64; 95%CI 1.21–2.22) (Table 2).

**Table 2:** Prevalence and prevalence ratios of isolated and combined exposure according to the occurrence of CMD, in health workers. State of Bahia, Brazil, 2012.

Variable	n	Prevalence of CMD (%)	PR	95%CI
Reference				
Social support = 0, high demand = 0	228	17.0	1.00	
Isolated exposures				
Social support = 0, high demand = 1	325	24.0	1.41	1.02–1.94
Social support = 1, high demand = 0	140	17.9	1.05	0.67–1.62
Combined exposures				
Social support = 1, high demand = 1	393	28.0	1.64	1.21–2.22
Expected combined effect^a^		24.9	1.46	
RERI^b^			0.18	
AP^c^			0.10	
S^d^			1.39	

CMD: Common mental disorders; PR: Prevalence ratio

^a^ Expected combined effect = P_01_ - P_00_ + P_10_ - P_00_ + P_00_ / PR_01_ - PR_00_ + PR_10_ - PR_00_ + PR_00_

^b^ Excess risk due to interaction (RERI) = PR_11_ - PR_01_ - PR_10_ + 1

^c^ Attributable proportion due to interaction (AP) = (PR_11_ - PR_01_ - PR_10_ + 1) / PR_11_

^d^ Synergy index (S) = (PR_11_ - 1) / (PR_01_ + PR_10_ - 2)

The difference between the expected prevalence for combined exposure (P = 24.9%) and the prevalence observed for the group (P = 28%) exceeded the occurrence of the outcome in 3.1%, according to the assumption of additivity. The association of the combined effect of high demand and low social support (PR = 1.64) was higher than what was estimated based on the additivity of the effects (PR = 1.46), resulting in RERI = 0.18. The attributable proportion due to interaction was 10.0% directed to the synergy of the effects (S = 1.39) (Table 2).

The data showed that 17% of the prevalence of the CMD corresponded to unknown exposures, which represent individuals who developed the disease even without being exposed to any of the factors investigated. On the other hand, 7% of the prevalence of the outcome was recorded in the situation of high demand and 0.9% in low social support in the workplace. Exposure to both factors was responsible for 11% of the excess of prevalence. The combined effect of the factors (EPR = 0.64) was greater than the sum of their isolated effects (EPR = 0.46). The relative difference between the estimates of the effects indicated a deviation of 39% of the expected behavior for the independent action of the factors (Table 3).

**Table 3:** Excess of prevalence and excess of prevalence ratios for isolated and combined effects of high demand and low social support in the workplace on the occurrence of CMD, in health workers. State of Bahia, Brazil, 2012.

Variable	n	Excess of prevalence^a^	Excess of prevalence ratio (EPR = PR - 1)		Relative difference [(A/B) - 1] (%)

			Observed (EPRO) (A)	Expected based on separated exposure^b^ (B)	
Reference					
Social support = 0, high demand = 0	228	-		-	
Isolated exposures					
Social support = 0, high demand = 1	325	7.0	0.41	-	
Social support = 1, high demand = 0	140	0.9	0.05	-	
Combined exposures					
Social support = 1, high demand = 1	393	11.0	0.64	0.46	39

CMD: Common Mental Disorders

^a^ Excess of prevalence ratio = (EP = P_exposure_ - P_no exposure_)

^b^ Excess of prevalence ratio expected based on separated exposure = EPRO_01_ + EPRO_10_

## DISCUSSION

The demographic profile of the studied population showed similarity with other investigations carried out with primary health care workers: young individuals, mostly female, with only one job, and working for a short time in the unit^13^. The findings for the psychosocial characteristics of the work showed that most of the workers were exposed to high psychological demands and low decision latitude, similar to studies conducted among health professionals^6^. This shows a troubling reality, as this work experience increases the predisposition of workers to negative health outcomes^4^. On the other hand, most workers reported high social support in the workplace, a psychosocial aspect able to protect them from the negative influences of stress on their psychological and physical well-being, acting as a protective factor^10^.

The prevalence of CMD among workers was lower when compared to research studies conducted with health workers in Brazil^3^. However, it presented a relatively high prevalence (affecting one worker every five studied) and, therefore, cannot be neglected. Health work, as it obeys the logic of human labor, requires a mental conception that precedes the execution of the work in itself^18^. In this sense, considering that the CMD will impose some level of impairment to the mental function of individuals, their occurrence is of great importance among health professionals. That is because their work is characterized by the peculiarity of their product, which is consumed the moment it is produced, reflecting the implementation of the activity in itself^20^. In addition, despite the findings on the quality of the care provided, they will significantly impact the well-being and quality of life of workers^6^.

The combined effect of high demand and low social support in the workplace showed higher occurrence of CMD than the expectation based on additivity, i.e., there are excessive events that characterize the synergism between the effects of these factors in the occurrence of the disease. The low decision latitude, the high psychological demand, and the low social support are an ideal scenario for adverse events among workers. On the other hand, high control, low demand, and high social support are the best work situation experienced and they contribute to promoting occupational health^16^ ^,^ ^17^.

The effects of the psychosocial aspects of the work on health have received considerable attention in recent years. The analysis of these aspects has been used successfully to predict the occurrence of mental disorders and other events related to health^26^. Evidence based on longitudinal studies shows that high levels of psychological demands and low control over work are predictive factors for mental disorders^7^. A study conducted with a population of workers in Sweden^8^ shows synergistic interaction between decision latitude and social support in the workplace for the occurrence of CMD.

The mechanisms that explain the association between stress at work and disease are based on the theory that chronic stress produces changes in the circadian rhythm of cortisol, which, in turn, implies in organic, emotional, and mental repercussions. In addition, stress at work can lead to the adoption of unhealthy behaviors by individuals, with equally negative consequences for the health. On the other hand, the buffer effect theory states that social connections bring health benefits by providing the resources needed to handle stress^9^. Thus, social support is a beneficial factor because it can reduce the perception of threats and it acts as an important environmental resource in the process of confronting adverse situations.

Social support is an essential psycho-social aspect in all sectors of work, but it is especially significant in the health work because of the relational character involved in the activities. It allows individuals to improve their ability to solve the problems presented by users and by the dynamics of the service, in their daily work, showing solidarity when faced with adverse situations, both witnessed and experienced. In addition, it enables the sharing of mutual commitments, in this case, the improvement of the health levels of the population cared for and the working conditions. It also contributes to job satisfaction and the feeling of recognition and appreciation for the effort undertaken with the health care provided^a^.

In primary health care, in addition to the aspects investigated, some factors can be mentioned as potentially causes of stress among workers, such as the growing number of temporary contracts, job insecurity, performance evaluation based on productivity, and lack of balance between private life and work. These factors are combined with the precarious conditions of life and health of the populations cared for, which increases the pressure on the workers and leads to greater occurrence of illness^11^ ^,^ ^19^. Therefore, in addition to the factors related to the psychosocial dimension of work, other factors must be evaluated among these workers.

This study has limitations. The interpretation of the associations between psychosocial aspects at work and CMD should be evaluated with caution. The information on both variables came from self-reports, which provides subjective data susceptible to bias. Because this is a cross-sectional study design, no causal relationships can be established. The simultaneous measurement of the exposures and the manifestation of the disease do not allow us to rule out alternative explanations for the associations found. In addition, there is a possibility of increased false-positive results, as workers with compromised health are more likely to report their work situation as being psychologically demanding, unlike healthy workers. Such a trend can lead to misclassification, which can result in both overestimation and underestimation of the true effect. There is still the possibility of healthy worker bias, as we have only included the participation of individuals who were in full exercise of their professional activities. Those who were eventually away, diseased by the work itself, were not part of the sample studied.

The results obtained strengthen the hypothesis of interaction between the psychosocial aspects of the work for the occurrence of CMD among health workers, directed to the synergy of the effects. Although similar investigations have not been identified for comparison, the literature points to plausibility for the associations found here. In this way, it is pertinent to consider intervention in at least some of the factors investigated here, considering greater emphasis on aspects related to the organization of the work. This study may also contribute to the reflection on the psychosocial aspects and occupational health in the perspective of the relational nature of the work, in which social support plays a central role.

## Footnotes

a Ferreira RC. Apoio social no trabalho e absenteísmo-doença em trabalhadores de enfermagem [dissertation]. Rio de Janeiro (RJ): Escola de Enfermagem Anna Nery da Universidade Federal do Rio de Janeiro; 2010.
